# Potential of the enzyme laccase for the synthesis and derivatization of antimicrobial compounds

**DOI:** 10.1007/s11274-023-03539-x

**Published:** 2023-03-01

**Authors:** Veronika Hahn

**Affiliations:** 1 Leibniz Institute for Plasma Science and Technology (INP), Felix-Hausdorff-Str. 2, 17489 Greifswald, Germany; 2 Institute for Microbiology, University of Greifswald, Felix-Hausdorff-Str. 8, 17489 Greifswald, Germany

**Keywords:** Antimicrobial compounds, Cross coupling, Cyclization, Laccase, White chemistry

## Abstract

Laccases [E.C. 1.10.3.2, benzenediol:dioxygen oxidoreductase] can oxidize phenolic substances, e.g. di- and polyphenols, hydroxylated biaryls, aminophenols or aryldiamines. This large substrate spectrum is the basis for various reaction possibilities, which include depolymerization and polymerization reactions, but also the coupling of different substance classes. To catalyze these reactions, laccases demand only atmospheric oxygen and no depletive cofactors. The utilization of mild and environmentally friendly reaction conditions such as room temperature, atmospheric pressure, and the avoidance of organic solvents makes the laccase-mediated reaction a valuable tool in green chemistry for the synthesis of biologically active compounds such as antimicrobial substances. In particular, the production of novel antibiotics becomes vital due to the evolution of antibiotic resistances amongst bacteria and fungi. Therefore, laccase-mediated homo- and heteromolecular coupling reactions result in derivatized or newly synthesized antibiotics. The coupling or derivatization of biologically active compounds or its basic structures may allow the development of novel pharmaceuticals, as well as the improvement of efficacy or tolerability of an already applied drug. Furthermore, by the laccase-mediated coupling of two different active substances a synergistic effect may be possible. However, the coupling of compounds that have no described efficacy can lead to biologically active substances by means of laccase. The review summarizes laccase-mediated reactions for the synthesis of antimicrobial compounds valuable for medical purposes. In particular, reactions with two different reaction partners were shown in detail. In addition, studies with in vitro and in vivo experimental data for the confirmation of the antibacterial and/or antifungal efficacy of the products, synthesized with laccase, were of special interest. Analyses of the structure–activity relationship confirm the great potential of the novel compounds. These substances may represent not only a value for pharmaceutical and chemical industry, but also for other industries due to a possible functionalization of surfaces such as wood or textiles.

## Introduction

Especially in view of an increasing number of multidrug-resistant microorganisms, there is a rising demand for antimicrobial compounds with a broad spectrum against a wide range of microorganisms and only few side effects. It has been estimated by the WHO that antibiotic-resistant microorganisms will cause about 10 million deaths by the year 2050 (WHO 2016). Such infectious diseases are a serious health concern and life-threatening in particular for elderly and immunocompromised persons. Thus, a continuous search for an environmentally friendly synthesis of novel antibiotics or the derivatization of known antibiotics is needed. In this regard, reactions mediated by enzymes may be an alternative towards chemical processes. The enzyme laccase is very promising for this purpose.

The laccase [E.C. 1.10.3.2, benzenediol: O_2_ oxidoreductase] was first discovered in exudates of the Japanese lacquer tree *Toxicodendron verniciflua* (earlier named as *Rhus vernicifera*) by Yoshida in 1883 (Yoshida 1883). Since then, more than 100 fungal laccases have been found in Ascomycota (e.g. *Aspergillus* species), Deuteromycota (e.g. *Botrytis* species), and especially in ligninolytic Basidiomycota. The white rot fungi belong to the latter group and include the genera *Trametes*, *Pycnoporus* and *Phanerochaete*, which turned out to be outstanding laccase producers (Hermann et al. 1983; Slomczynski et al. 1995; Srinivasan et al. 1995; Eggert et al. 1996; Yaver et al. 1996; Han et al. 2021). In particular, the high amount of laccase and the comparatively simple isolation of the enzyme from the supernatant of the culture medium make white rot fungi and their laccases valuable for biosynthetic processes, e.g. the production of antibiotics.

Laccases belong to the ligninolytic enzyme system together with lignin and manganese peroxidase as well as versatile (hybrid/manganese-lignin) peroxidase (Leonowicz et al. 2001; Kumar and Chandra 2020). Unlike peroxidases, laccases do not require hydrogen peroxide as a cofactor. Bacteria (Hullo et al. 2001; Sharma et al. 2007), insects (Dittmer et al. 2004; Asano et al. 2019), algae (Otto and Schlosser 2014), molluscs (Luna-Acosta et al. 2017), and sponges (Li et al. 2015) produce also laccases or laccase-like enzymes. Even for humans a *LACC1* gene was detected with a C-terminus homologous to bacterial oxidoreductases and laccases (Assadi et al. 2016). In general, descriptions about the utilization of non-fungal laccases for the synthesis of biologically active compounds are rare. Nevertheless, the formation of potentially antimicrobial substances by the CotA-laccase of the bacterium *Bacillus subtilis* has been described (Sousa et al. 2014, 2018).

The physiological functions are as diverse as the sources of laccases (Janusz et al. 2020). On the one hand, laccases catalyze catabolic reactions, e.g. during lignin degradation by white rot fungi (Leonowicz et al. 2001); on the other hand, laccases in plants, insects, bacteria, and fungi are involved in various polymerization reactions such as for morphogenesis.

An important advantage of laccases for the physiological function and, in particular, for synthetic purposes is the broad substrate spectrum. The enzyme oxidizes, for example, di- and polyphenols, aminophenols, methyl- or methoxy-substituted phenols and aryldiamines (Bollag et al. 1988; Keilin and Mann 1939; Yaropolov et al. 1994; d'Acunzo et al. 2002; Giurg et al. 2007). The substrates includes compounds such as salicylic acid esters (Ciecholewski et al. 2005), vanillic and syringic acid (Leonowicz et al. 1984), 2-hydroxydibenzofuran (Jonas et al. 2000), and *ortho*- (Hosny and Rosazza 2002; Mikolasch et al. 2008c) and *para*-dihydroxylated (Anyanwutaku et al. 1994; Manda et al. 2005) aromatic compounds.

**Fig. 1:**
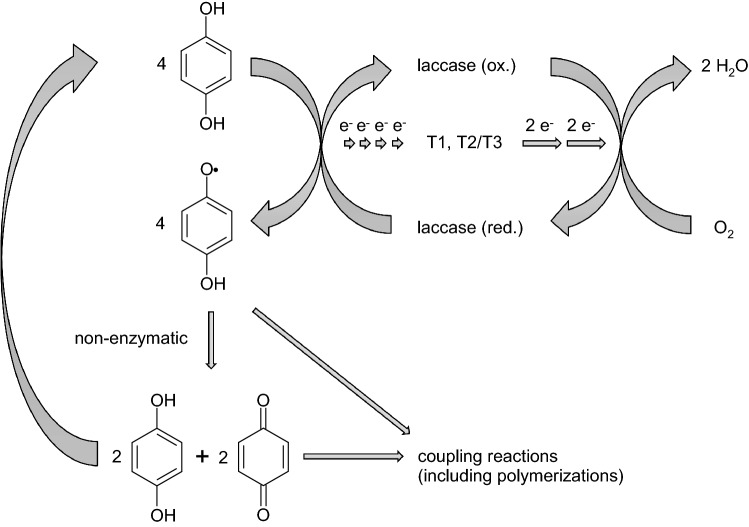
Catalytic cycle of laccase-mediated oxidation of substrates and subsequent reactions (modified according to Thurston 1994; Solomon et al. 2001, 2008; Claus 2003; Baldrian 2006)

Laccases belong to the group of blue multicopper oxidases together with ascorbate oxidase (from plants) and ceruloplasmin (from vertebrates; Solomon et al. 1996; Claus 2003). The oxidation of substrates by laccase is a one-electron reaction resulting in the formation of a free radical (Solomon et al. 1996, 2001, 2008). One electron from each of four substrate molecules is transferred to the enzyme. Oxygen serves as electron acceptor. Four electrons are transferred to oxygen, forming two molecules of water. The catalysis takes place via four copper atoms, which are classified into different types according to their electronic properties and can be distinguished based on their spectroscopic characteristics. The copper atom located in a so-called type 1 binding site (T1), the copper atom in the type 2 (T2), and the two copper atoms in the type 3 binding site (T3) are involved in the catalytic process (Fig. 1). T2 and T3 are spatially close to each other and form the so-called trinuclear copper center. The oxidation of the substrate molecules takes place at T1 by a stepwise transfer of four electrons. The electrons are then transferred to T2/T3. The reduction of molecular oxygen to water occurs at T3 by a two-step transfer of four electrons, whereby the laccase is reoxidized. The radical formed by the laccase-mediated reaction, as well as the thereof non-enzymatically formed products can undergo coupling reactions that can lead to the synthesis of polymers (Claus 2003, 2004). The resulting hydroquinonoid compound represents a laccase substrate and is again subject to oxidation, although a participation in coupling reactions cannot be ruled out either.

In addition to the catalytic reaction, the four copper atoms of the catalytic center are also responsible for other properties, e.g. the color and the possibility for an inhibition of the laccase. Thus, laccases can be inhibited, for example, by fluoride, chloride, cyanide, azide and hydroxyl anions which bind to T2/T3 and thereby prevent electron transfer (Keilin and Mann 1939; Xu 1996, 1997; Johannes and Majcherczyk 2000).

Structural differences of laccases may also account for different redox potentials of individual laccases (Piontek et al. 2002). Thus, laccases are classified into low potential (+ 0.5 V, determined against a normal hydrogen electrode; e.g. *Myceliophthora thermophila*) and high potential (+ 0.7 to + 0.8 V; e.g. *Pycnoporus cinnabarinus*, *Trametes* spec.) according to their redox potential (Xu 1996; Xu et al. 1998; Li et al. 1999). Xu (1996) described that the redox potential difference between enzyme and substrate has an influence on enzymatic oxidation. Laccases can only oxidize substrates whose redox potentials are below their own (Xu et al. 2000). This explains why, for example, *ortho*- and *para*-dihydroxylated aromatic compounds are particularly good laccase substrates. These compounds have a low redox potential (+ 0.5 to + 0.6 V). Contrastly, *meta*-diphenols have a redox potential between + 0.8 and + 1.1 V and thus cannot be oxidized by laccases or can only be oxidized with difficulty (Mai et al. 2001). In addition to the redox potential, steric hindrances also play a role in the oxidation ability of a compound. For example, Tadesse et al. (2008) showed that 2,4,6-tritertiary butylphenol and 2,6-diisopropylphenol were oxidized in different amounts despite the same redox potential, which was attributed to steric hindrances of the substituents in binding the substrates in the enzymatic pocket.

Mediators can be used to overcome high redox potentials or steric problems (Tadesse et al. 2008). Mediators are compounds with a low molecular weight that can be used to oxidize substances indirectly by the laccase. In this process, the laccase first oxidizes the mediator, which diffuses away from the enzymatic pocket. Afterwards, the oxidized mediator is able to oxidize the substance (d'Acunzo et al. 2002; Claus 2003; Mogharabi and Faramarzi 2014; Bassanini et al. 2021). Bourbonnais and Paice (1990) showed that in the presence of 2,2'-azino-bis-(3-ethylbenzothiazoline-6-sulfonic acid) diammonium salt (ABTS), non-phenolic lignin model components, such as veratryl alcohol, can also serve as laccase substrates. ABTS as well as 2,2,6,6-tetramethylpiperidin-1-yloxy (TEMPO) and 1-hydroxybenzotriazole (HBT) are synthetic mediators. Natural mediators include malonate or oxalate. The mode of action for *N*-hydroxy mediators, e.g. TEMPO and HBT comprises the oxidation of these compounds to radicals that react with the hydroxyl group of the substrate. Thereby, the hydrogen of the hydroxyl group is transferred to the radical forming hydroxylamine and in turn the substance is oxidized (d’Acunzo et al. 2002; Mogharabi and Faramarzi 2014; Obleser et al. 2022).

The utilization of laccase alone or in combination with mediators has been described in multienzymatic and also chemoenzymatic procedures for the synthesis of organic compounds including pharmaceutical building blocks and biologically active substances (for reviews please refer to Mogharabi and Faramarzi 2014; Bassanini et al. 2021). Syntheses catalyzed by laccase-mediator-systems were not subject of the present review. Laccases without or with mediators can catalyze, the bleaching of wood (Balakshin et al. 2001; Valls and Roncero 2009), the removal of dyes from wastewater, e.g. in the textile industry (Campos et al. 2001; Wesenberg et al. 2003), and the functionalization of lignin (grafting), e.g. to increase the solubility (for reviews please refer to Widsten and Kandelbauer 2008 and Agustin et al. 2021). In these areas, there is a great interest in laccase as a "green" i.e. environmentally friendly catalyst that requires only oxygen and whose reaction produces only water as a by-product (Riva 2006).

### Reaction possibilities and utilization in white biotechnology

**Fig. 2:**
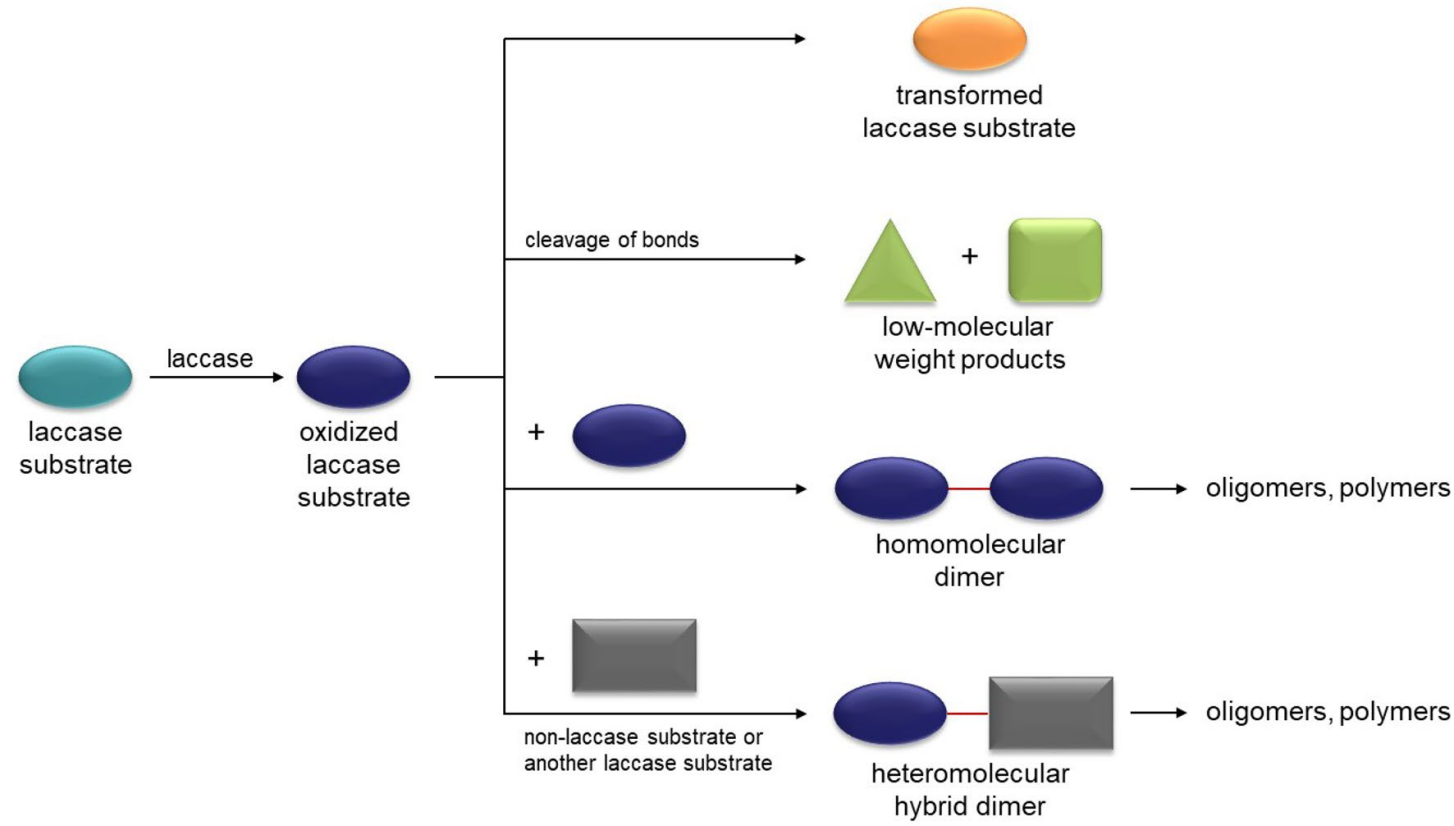
Possibilities for laccase-mediated reactions

The oxidation of laccase substrates (e.g. aromatic compounds with one or more hydroxyl groups) leads to the formation of free radicals (Solomon et al. 1996; Claus 2004). Different mesomeric structures can be described through the delocalization of π-electrons. In this way, numerous aromatic laccase substrates can be "activated". The radical may undergo homo- and heteromolecular reactions. The homomolecular reaction proceeds in an assay which contains only one reactant and results in transformed/modified or cleaved laccase substrates as well as coupling reactions forming di-, oligo- or polymers (Fig. 2). In contrast, the assay for the heteromolecular reaction contains different reaction partners. The possible reaction partners include compounds that can be oxidized by the laccase and substances that cannot be oxidized. Particularily, the latter case allows coupling reactions with hundreds of non-laccase substrates and makes the enzyme laccase indispensable for white biotechnology.

In this way, the formation of homo- and heteromolecular products is accessible. The reaction partner(s) can be linked one or more times depending on the number and type of substituents as well as other parameters such as the reaction time, leading to the formation of di-, tri- or polymers. The linkage of the molecules and thus the type of bond depends on the reaction partners. The homomolecular reaction leads to C–C (Jonas et al. 2000), C=C (Simmons et al. 1989) and also C–O bonds (Aruwa et al. 2021). The heteromolecular hybrid molecules can be formed for phenolic compounds via C–C (Cannatelli and Ragauskas 2015), C=C (Simmons et al. 1989) and C–O bonds (Bollag and Liu 1985) or in the reaction with aromatic amines by C–N (Manda et al. 2005) or C=N (Tatsumi et al. 1994) bonds.

Especially in the formation of C–N bonds it is assumed that in the course of the laccase-catalyzed reaction cation radicals are formed, which subsequently react with the amino group of the reaction partner via a nucleophilic addition (Michael addition, 1,4-addition; Niedermeyer et al. 2005). In addition to the bond types presented, C–S bonds are also possible (Benfield et al. 1964; Wellington et al. 2012). In the case of ring formations, multiple bonds are formed (Bhalerao et al. 1994; Hajdok et al. 2007). Reactions with solvents result, for example, in methoxylations (Manda et al. 2007). Summaries of laccase-catalyzed homo- and heteromolecular reactions with different focal points have been published previously (Riva 2006; Mikolasch and Schauer 2009; Kudanga et al. 2017; Sousa et al. 2021; Cardullo et al. 2022).

In addition to the coupling reactions, also the cleavage of compounds (Kawai et al. 1988) or substituents, e.g. dechlorination (Leontievsky et al. 2001) or decarboxylation (Agematu et al. 1993b), is accessible with the enzyme laccase whereby the cleavage products may also be involved in synthesis reactions (Hahn et al. 2014).

The advantage of the laccase-mediated process for the coupling of substances, known as combinatorial biochemistry, over chemical synthesis methods lies primarily in the use of mild, environmentally friendly reaction conditions, such as the use of room temperature and atmospheric pressure, and the avoidance of organic solvents. In addition, the production of a wide range of substances in a short time and the conversion of even sensitive natural and biologically active substances is possible. Compounds can be synthesized that are rarely found in nature or that must first be isolated from natural sources at great expense. By conserving natural resources, enzymatic synthesis also offers a way to contribute to sustainability.

The use of an enzyme as catalyst may be regarded as a disadvantage but the laccase process is a catalytic cycle, whereas chemical catalysts are needed in stoichiometric amounts that means these catalysts are consumed in the course of the reaction time.

The synthesis and derivatization of biologically active agents or substances with basic drug structures represent an innovative application for the laccase. For this reason, the following examples will focus on the laccase-mediated production of potentially pharmaceutically useful substances.

### Synthesis of antimicrobial compounds

**Fig. 3:**
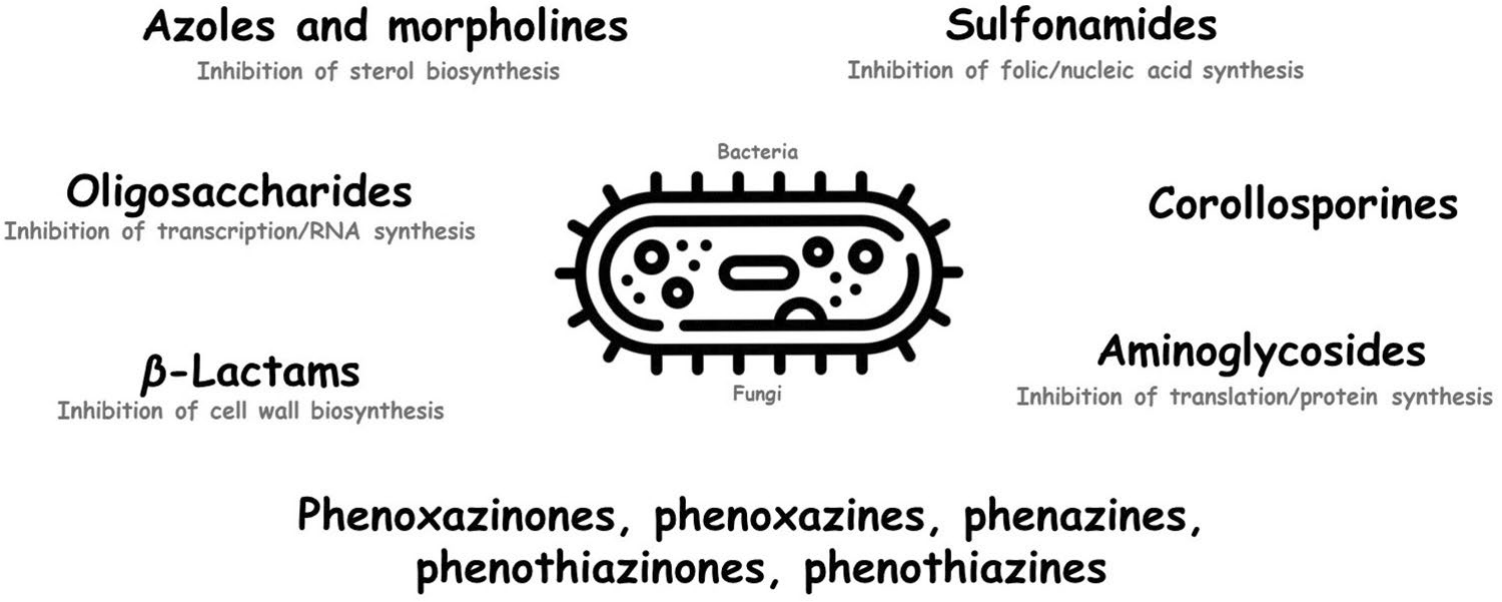
Substances that can be synthesized by laccase-mediated processes (including targets of antimicrobial compounds on bacteria and fungi)

In the past years different strategies have been pursued regarding the synthesis of biologically active compounds. Thus, already described pharmaceuticals or biologically active substances may undergo laccase-mediated modification or can be derivatized with also active or inactive compounds (Table 1). The derivatization or coupling of biologically inactive compounds forming active products is also conceivable. These approaches may not only result in an enhancement of the original pharmaceutical efficacy but also the generation of novel properties such as higher tolerability, a different efficacy than the parent compound (e.g. parent compound was antioxidative, product has antibacterial activity) or a broader spectrum (e.g. parent compound was only antibacterial, product has antibacterial and antifungal activity) is possible. The review shows the laccase-mediated syntheses of different antimicrobial or potentially antimicrobial compounds (Fig. 3). In particular, studies with antimicrobial susceptibility data were described. Only these in vitro and in vivo assays allow analyses of the relationship between chemical structures and biological activity of the products synthesized by laccase.

**Table 1:** Possible applications of laccase for the synthesis and derivatization of biologically active substances in the context of homomolecular (i.e. assays with only one reactant) and heteromolecular reactions (i.e. assays with different reactants); References were ordered according to the appearance in the text

Substance I (non-laccase substrate or another laccase substrate)	Activity of substance I	Substance II (laccase substrate) and activity	Origin of laccase	Reaction product	Yield [%]	Test system	Activity of product in comparison to parent compound(s)^*a,b*^	Tested microorganisms^*c*^	Tested multidrug-resistent microorganisms	References
β**-Lactams**										
No partner	n. a.	Penicillin X methyl ester; substance: antibacterial	*Coriolus versicolor* company: n. d.	C–C dimer C–O dimer C–O C–C dimer	n. d.	Determination of MIC by broth dilution	0; (antibacterial)	n. d.		(Agematu et al. 1993c)
No partner	n. a.	Penicillin X pivaloyloxy-methyl ester; substance: antibacterial	*Coriolus versicolor* company: n. d.	C–C dimer	n. d.	Determination of MIC by broth dilution	1; antibacterial after incubation with esterase	*B. subtilis*, *E. coli*, *M. luteus, S. aureus, S. epidermidis*		(Agematu et al. 1993c)
No partner	n. a.	7-(4-Hydroxyphenyl-acetamido)-cephalosporanic acid; substance: antibacterial	*Coriolus versicolor* IFO 9791 company: n. d.	Spiro-epoxide (7-[1-Oxa- spiro(2.5) octa-6-oxo- 4,7-diene-2-car- boxamido]- cephalosporanic acid)	n. d.	Determination of MIC by broth dilution	1; antibacterial	*B. subtilis*, *E. coli*, *K. pneumoniae, M. luteus, Se. marcescens, S. aureus, P. vulgaris, Ps. aeruginosa*		(Agematu et al. 1993a)
Amoxicillin Ampicillin	Antibacterial	2,5-Dihydroxybenzamide 2,5-Dihydroxybenzoic acid ethyl ester 2,5-Dihydroxybenzoic acid methyl ester 2,5-Dihydroxy-*N*-(2-hydroxyethyl)-benzamide	*Trametes* spec. (laccase C; ASA Spezialenzyme GmbH, Wolfenbüttel, Germany)	C–N dimer	n. d.	Disk diffusion test (on Mueller–Hinton-agar II)	1 to 2; antibacterial	*B. megaterium, B. subtilis*, *En. faecalis*, *E. coli*, various *S. aureus* and *S. epidermidis* strains, *Ps. aeruginosa, Ps. maltophilia*	Various strains isolated from patients e.g. MRSA, *En. faecalis* (vancomycin-resistant)	(Mikolasch et al. 2006)
						Neutral red uptake assay using FL-cells	Products: no or weak cytotoxicity up to 100 µg/ml			
						*Staphylococcus aureus*-infected, immune suppressed mouse model	2; antibacterial			
						Disk diffusion test (on Mueller–Hinton-agar II) with *Staphylococcus aureus* (stability against *β*-lactamase-1 and -2)	2 to 3—for one product shown; stable against *β*-lactamase-1 and -2			
						Determination of octanol–water partition coefficient (log D)—Absorption	3			
Cefadroxil Cefalexin Cefaclor Loracarbef	Antibacterial	2,5-Dihydroxybenzamide 2,5-Dihydroxybenzoic acid ethyl ester 2,5-Dihydroxybenzoic acid methyl ester 2,5-Dihydroxy-*N*-(2-hydroxyethyl)-benzamide	*Myceliophthora thermophila* (NovoNordisk A/S, Bagsværd, Denmark) *Trametes* spec. (laccase C; ASA Spezialenzyme GmbH, Wolfenbüttel, Germany)	C–N dimer	74–88	Disk diffusion test (on Mueller–Hinton-agar II)	1 to 3; antibacterial	*B. megaterium, B. subtilis*, *En. faecalis*, *E. coli*, various *S. aureus* and *S. epidermidis* strains, *Ps. aeruginosa, Ps. maltophilia*	Various strains isolated from patients e.g. MRSA, *En. faecalis* (vancomycin-resistant)	(Mikolasch et al. 2007)
						Neutral red uptake assay using FL-cells	Products: no or weak cytotoxicity up to 100 µg/ml			
						*Staphylococcus aureus*-infected, immune suppressed mouse model	2; antibacterial			
Amoxicillin Ampicillin Cefadroxil Loracarbef	Antibacterial	Catechol 3-Methylcatechol 4-Methylcatechol; substances: in part antibacterial	*Trametes* spec. (laccase C; ASA Spezialenzyme GmbH, Wolfenbüttel, Germany)	C–N dimer	27–83	Disk diffusion test (on Mueller–Hinton-agar II)	1 to 3; antibacterial	*En. faecalis*, *E. coli*, various *S. aureus* and *S. epidermidis* strains	Various strains isolated from patients e.g. MRSA, *En. faecalis* (vancomycin-resistant)	(Mikolasch et al. 2008c)
						Neutral red uptake assay using FL-cells	2 to 3; cytotoxic			
						*Staphylococcus aureus*-infected, immune suppressed mouse model	1; antibacterial			
Amoxicillin Ampicillin Cefaclor Cefadroxil Cefradin Cefalexin Loracarbef	Antibacterial	2,5-Dihydroxybenzoic acid 2,5-Dihydroxyphenylacetic acid and esters thereof 3,4-Dihydroxybenzoic acid 3,4-Dihydroxyphenylacetic acid, 3-(3,4-Dihydroxy-phenyl)-propionic acid 3,5-Dimethoxy-4-hydroxybenzoic acid 3-(3,5-Dimethoxy-4-hydroxyphenyl)-2-propenoic acid, 4-Hydroxy-3-methoxybenzoic acid; 2,5-Dihydroxyphenylacetic acid used for product synthesis: not antibacterial	*Myceliophthora thermophila* (Novozymes A/S, Bagsværd, Denmark) *Pycnoporus cinnabarinus* (own preparation) *Trametes* spec. (laccase C; ASA Spezialenzyme GmbH, Wolfenbüttel, Germany)	C–N dimer	34–61	Disk diffusion test (on Mueller–Hinton-agar II)	1 to 3; antibacterial	*En. faecalis*, various *S. aureus* and *S. epidermidis* strains	Various strains isolated from patients e.g. MRSA, *En. faecalis* (vancomycin-resistant)	(Mikolasch et al. 2012)
						Neutral red uptake assay using FL-cells	2; no cytotoxicity			
						*Staphylococcus aureus*-infected, immune suppressed mouse model	1 to 2; antibacterial			
Amoxicillin Ampicillin Cefaclor Cefadroxil Cefradin Cefalexin Loracarbef	Antibacterial	38 dihydroxylated aromatic compounds (1,2- or 1,4-hydroquinonoid substances) e.g. 2,3-Dimethyl-hydroquinone Methylhydroquinone; substances: in part antibacterial	*Myceliophthora thermophila* (Novozymes A/S, Bagsværd, Denmark)	C–N dimer	13–80	Disk diffusion test (on Mueller–Hinton-agar II)	1 to 3; antibacterial	*B. megaterium, B. subtilis*, *En. faecalis*, *E. coli*, various *S. aureus* and *S. epidermidis* strains	Various strains isolated from patients e.g. MRSA, *En. faecalis* (vancomycin-resistant)	(Mikolasch et al. 2016)
						Determination of MIC by broth microdilution	1 to 3; antibacterial	*S. aureus* strains	Multidrug-resistant *S. aureus*	
						Neutral red uptake assay using FL-cells	Products: no or weak cytotoxicity up to 100 µg/ml; (3; in comparison to laccase substrate)			
						*Staphylococcus aureus*-infected, immune suppressed mouse model	1 to 2; antibacterial			
						Disk diffusion test (on Mueller–Hinton-agar II) with *Staphylococcus aureus* (stability against *β*-lactamase-1–4)	1 to 3; stable against β-lactamases			
6-Aminopenicillanic acid, 7-Aminocephalosporanic acid 7-Aminodesacetoxycephalo-sporanic acid	Not antibacterial	2,5-Dihydroxybenzoic acid methyl ester 2,5-Dihydroxy-*N*-(2-hydroxyethyl)-benzamide; substances: not antibacterial	*Trametes* spec. (laccase C; ASA Spezialenzyme GmbH, Wolfenbüttel, Germany)	C–N dimer	63–73	Disk diffusion test (on Mueller–Hinton-agar II)	3; antibacterial	*S. aureus* strains, *S. epidermidis*	Various strains isolated from patients e.g. MRSA	(Mikolasch et al. 2020)
						Neutral red uptake assay using FL-cells	Products: no cytotoxicity up to 100 µg/ml			
**Sulfonamides**										
Dapsone Sulfamerazine Sulfanilamide	Antibacterial	2,5-Dihydroxyacetophenone 2,5-Dihydroxybenzoic acid methyl ester 2,5-Dihydroxy-1,4-benzenediacetic acid 2,5-Dihydroxy-*N*-(2-hydroxyethyl)-benzamide 2,5-Dihydroxyphenylacetic acid; substances: in part antibacterial	*Trametes* spec. (laccase C; ASA Spezialenzyme GmbH, Wolfenbüttel, Germany)	C–N dimers C–N, C–N trimers	10–93	Disk diffusion test (on Mueller–Hinton-agar II)	3; antibacterial	*S. aureus* strains, *S. epidermidis*	Various strains isolated from patients e.g. MRSA	(Mikolasch and Hahn 2021)
						Neutral red uptake assay using FL-cells	2; no cytotoxicity up to 100 µg/ml			
**Oligosaccharides**										
Mithramycin (Plicamycin)	Antibacterial, antitumor	Hydroquinone	*Polyporus anceps* company: n. d.	C–C dimer	n. d.	*In vivo* P388 leukemia antitumor assay in mice	1 in comparison with mithramycin; (antitumor)	n. a.	n. a.	(Anyanwutaku et al. 1994)
**Aminoglycosides**										
Gentamicin Glucosamin Kanamycin Tobramycin	Antibacterial	2,5-Dihydroxy-*N*-(2-hydroxyethyl)-benzamide; substance: not antibacterial	*Myceliophthora thermophila* (Novozymes A/S, Bagsværd, Denmark) *Pycnoporus cinnabarinus* (own preparation)	C–N C–O dimer	n. d.	Disk diffusion test (on Mueller–Hinton-agar II)	1 to 3; antibacterial	Various *S. aureus* and *S. epidermidis* strains, *S. haemolyticus*	Various strains isolated from patients e.g. MRSA	(Mikolasch et al. 2022)
						Neutral red uptake assay using FL-cells	2; no cytotoxicity up to 100 µg/ml			
						*Staphylococcus aureus*-infected, immune suppressed mouse model	1 to 2; antibacterial			
**Corollosporines**										
* N*-Analogous corollosporine derivatives	In part anti-bacterial	2,5-Dihydroxybenzoic acid methylester 2,5-Dihydroxy-*N*-(2-hydroxyethyl)-benzamide 4-Methylcatechol; 2,5-Dihydroxybenzoic acid derivatives: not antibacterial	*Trametes* spec. (laccase C) (ASA Spezial-enzyme GmbH, Wolfenbüttel, Germany)	C–N dimer	80–94	Disk diffusion test (on Mueller–Hinton-agar II)	2 to 3; antibacterial	*B. subtilis*, *C. maltosa, E. coli*, *S. aureus* strains, *S. epidermidis*, *S. haemolyticus, Ps. aeruginosa*	Various strains isolated from patients e.g. MRSA	(Mikolasch et al. 2008a)
**Azoles and morpholines**										
Morpholine 4-Morpholinoaniline	Not anti-microbial	2,5-Dihydroxybenzoic acid ethyl ester 2,5-Dihydroxybenzoic acid methyl ester 2,5-Dihydroxy-*N*-(2-hydroxyethyl)-benzamide 1,4-Dihydroxynaphthaline 2,3-Dimethylhydroquinone Hydroquinone Methylhydroquinone; substances: in part antimicrobial	*Myceliophthora thermophila* (Novozymes A/S, Bagsværd, Denmark)	C–N dimers (in part with involvement of C–O) C–N, C–N trimer	19–78	Disk diffusion test (on nutrient agar)	1 to 2; antimicrobial	*B. subtilis*, *C. maltosa, E. coli*, *S. aureus, Ps. aeruginosa*	n. d.	(Hahn et al. 2009)
						Determination of MIC by broth microdilution	1 to 3; antifungal	*C. maltosa, C. albicans*	n. d.	
						Neutral red uptake assay using HTB-9-cells	1 to 3; cytotoxic			
1-Aminobenzotriazole	Not anti-microbial	2,5-Dihydroxybenzoic acid ethyl ester 2,5-Dihydroxybenzoic acid methyl ester; substances: not antimicrobial	*Myceliophthora thermophila* (Novozymes, Bagsværd, Denmark)	C–N dimer oligomers	32–34	Disk diffusion test (on nutrient agar)	2 to 3; antimicrobial	*B. subtilis*, *C. maltosa, E. coli*, *S. aureus, Ps. aeruginosa*	n. d.	(Hahn et al. 2010b)
						Determination of MIC by broth microdilution	2 to 3; antifungal	*C. maltosa*	n. d.	
						Neutral red uptake assay using HTB-9-cells	3; cytotoxic			
2-Mercaptobenzoxazole 2-Mercaptobenzothiazole 2-Mercaptothiazoline	n. d.	3-Methoxycatechol and further catechols	*Agaricus bisporus* (ASA Spezial-enzyme GmbH, Wolfenbüttel, Germany)	C-S dimer	74–96	Determination of MIC	Product: antimicrobial	*C. albicans, En. faecium*, *S. aureus, Ps. aeruginosa*	n. a. in part clinical isolates	(Adibi et al. 2011; Abdel-Mohsen et al. 2014)
						Chemiluminescence assay, spectrophotometric free radical-scavenging test	n. d. Products: antioxidative			
**Phenoxazinones, phenoxazines, phenazines, phenothiazinones, phenothiazines**										
No partner	n. a.	3-Hydroxyanthranilic acid (2-amino-3-hydroxybenzoic acid)	*Coriolus hirsutus* (*Trametes hirsuta*; Calbiochem, San Diego, CA, USA) *Pycnoporus cinnabarinus* (own preparation)	C = N C–O dimer: phenoxazinone (cinnabarinic acid (2-amino-3-oxo-3*H*-phenoxazine-1,9-dicarboxylic acid))	n. d.	Determination of MIC by broth dilution	Product: antibacterial	*B.·subtilis, E. coli, Ps. aeruginosa S. aureus, K. pneumoniae, Sa. enteritidis, Str.* spec. group B, G, D, F	n. d.	(Eggert et al. 1995; Eggert 1997)
No partner	n. a.	3-Amino-4-hydroxybenzen-sulfonic acid	*Cerrena unicolor* (own preparation)	C = N C–O dimer: phenoxazinone	60; optimized: nearly 100%	Agar diffusion test (on lactose broth agar)	Product: not antibacterial up to 10 mg/ml	*S. aureus, E. coli*	n. d.	(Forte et al. 2010; Polak et al. 2016)
						Chemiluminescence assay, spectrophotometric free radical-scavenging test	1; antioxidative			
5-Aminonaphthalene-sulfonic acid 2-Aminonaphthalene-sulfonic acid 4-Aminonaphthalene-sulfonic acid	n. d.	2-Amino-3-methoxybenzoic acid	*Cerrena unicolor* (own preparation)	C–N dimer with subsequent formation of C = N dimer: phenazine	19–27	Agar diffusion test (on Mueller–Hinton-agar); Determination of MIC and MBC by broth dilution	Products: antimicrobial	*S. aureus, E. coli*	n. d.	(Polak et al. 2020)
						Chemiluminescence assay	Products: antioxidative			

*FL* Human amniotic epithelial cell line, *HTB-9* Human urinary bladder carcinoma cell line (ATCC 5637 - American Type Culture Collection), *MBC* Minimum bactericidal concentration, *MIC* Minimum inhibitory concentration, *MRSA* Methicillin-resistant *S. aureus*, *n. a.* not applicable, *n. d.* not determined/no data shown

^a^In brackets: potential/proposed activity without experimental data

^b^The synthesized compounds are compared with the parent compounds regarding the efficacy. In this context, the newly synthesized substance compared to the parent compound(s) may: (3) have a higher activity, (2) have the same, (1) a lower or (0) no activity

^c^*B. Bacillus, C. Candida, E. Escherichia, En. Enterococcus, K. Klebsiella, M. Micrococcus, S. Staphylococcus, Sa. Salmonella, Se. Serratia, Str. Streptococcus, P. Proteus, Ps. Pseudomonas*

### β-Lactams

Beta-lactam antibiotics inhibit the synthesis of the peptidoglycan layer of bacterial cell walls by binding to and inhibiting penicillin-binding proteins, a group of d-alanyl-d-alanine transpeptidases. This results in an impaired cell division (Miyachiro et al. 2019).

**Fig. 4:**
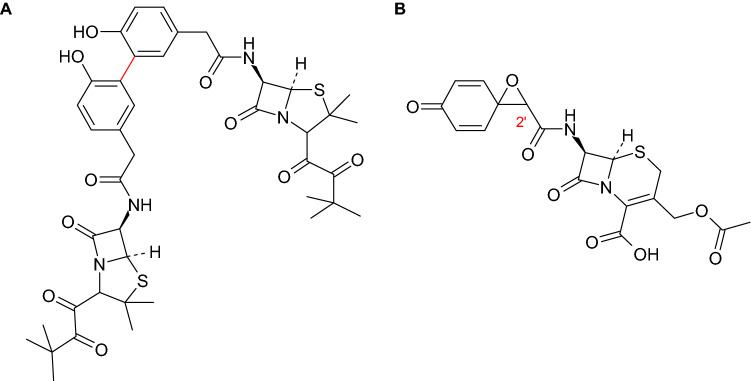
**A** Penicillin X pivaloyloxymethylester dimer (Agematu et al. 1993c) and **B** diastereomeric product 7-[1-oxaspiro(2.5)octa-6-oxo-4,7-diene-2-carboxamido]-cephalosporanic acid (Agematu et al. 1993a)

Agematu et al. (1993a, c) described the most prominent examples for laccase-mediated homomolecular reactions with biologically active compounds. This comprised the dimerization of antibiotics by laccase (Agematu et al. 1993c). Penicillin X esters were used as reagents for laccase-catalyzed reactions due to the higher stability and easier isolation of the dimers, compared with the reaction products of penicillin X sodium salt. The dimers of penicillin X methyl ester showed low antibacterial activity. In contrast, the *ortho*-*ortho* coupling product formed from penicillin X pivaloyloxymethyl ester represented a precursor (prodrug) of the penicillin X dimer (Fig. 4A). Thus, the pivaloyloxymethyl ester group was hydrolyzed in the presence of an esterase allowing the active portion of the molecule to inhibit the bacterial strains. However, the minimum inhibitory concentration was lower than that of penicillin X sodium salt.

The laccase-mediated reaction of a cephalosporanic acid derivative studied by Agematu et al. (1993a) resulted in the formation of a spiro-epoxide (Fig. 4B). The synthesized epoxide consisted of two diastereomers, which differed in the configuration at C-2' and the antibacterial activity. However, both substances showed lower efficacy than the parent compound.

In particular, the coupling of different substance classes by laccase enables the synthesis of a large number of organic hybrid molecules. These reactions result not only in heteromolecular dimers but also in oligomers. The coupling or derivatization of biologically active ingredients or basic structures of drugs allows the development of novel active compounds and drugs, as well as the improvement of the efficacy or even tolerability of an already applied drug. By laccase-mediated coupling of two different biologically active substances to a new compound various effects are conceivable.

Heteromolecular reactions are an advance over homomolecular reactions presented in the previous section. However, the coupling of compounds that have no described activity or that represent the basic structure of active substances can also lead to biologically active compounds by means of laccase.

For the synthesis of hybrid molecules, two different substances are incubated together with the laccase, whereby usually only one substance represents the laccase substrate.

The laccase-catalyzed synthesis of heteromolecular hybrid dimers resulting from the reaction of *β*-lactam antibiotics with dihydroxylated aromatic compounds was extensively studied by Mikolasch et al. (2006, 2007, 2008c, 2012, 2016) and Mikolasch (2019). The basic structure of penicillins, cephalosporins, and carbacephems is the *β*-lactam ring. This ring is connected with a five-membered thiazolidine ring in case of penicillins or a six-membered ring in case of carbacephems. Cephalosporins contain sulfur in the six-membered ring. In addition, the used *β*-lactam antibiotics possessed a free amino group, which predisposes them to laccase-catalyzed derivatization (Table 2). Thus, the formation of C–N bonds occurred between the amino group of the antibiotic and the corresponding quinone from *para*- (e.g. 2,5-dihydroxy-*N*-(2-hydroxyethyl)-benzamide) or *ortho*- (e.g. 3-methylcatechol) dihydroxylated aromatic compounds resulting in heteromolecular dimers.

**Table 2:**
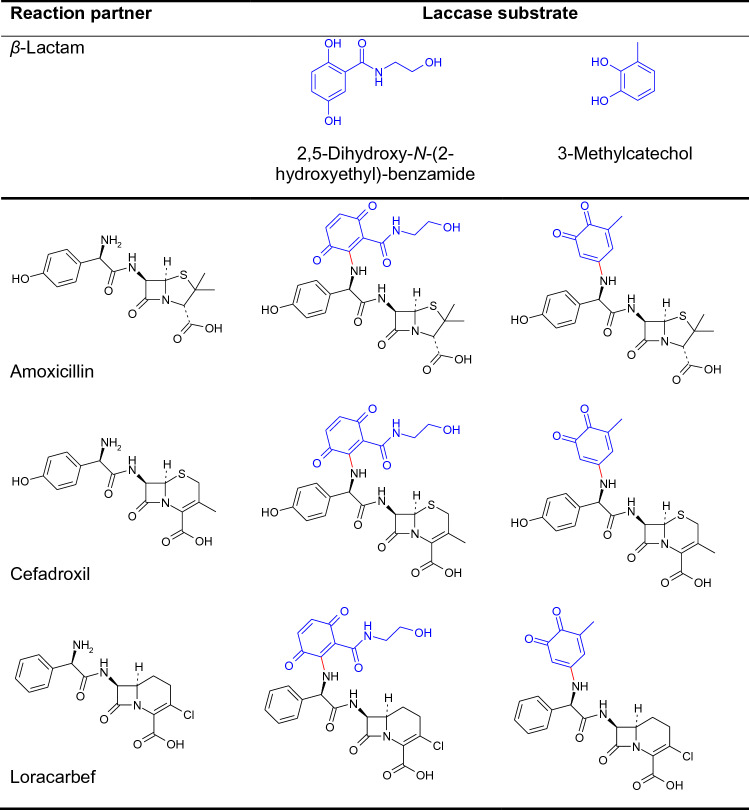
Heteromolecular dimers of laccase-mediated reactions of the *β*-lactam antibiotics amoxicillin (penicillin), cefadroxil (cephalosporin) or loracarbef (carbacephem) and 2,5-dihydroxy-*N*-(2-hydroxyethyl)-benzamide or 3-methylcatechol (Mikolasch et al. 2006, 2007, 2008c; Mikolasch 2019)

The resulting penicillin, cephalosporin or carbacephem hybrid dimers showed medium to high antibacterial efficacy against various microorganisms, including multidrug-resistant *Staphylococcus* and *Enterococcus* strains (Mikolasch et al. 2006, 2007, 2008c, 2012, 2016). The penicillin hybrid dimers resulted in a mostly similar or higher antimicrobial efficacy than the cephalosporin and carbacephem products (Mikolasch et al. 2006, 2007, 2008c, 2012, 2016) which was at least partly in accordance with the activity of the respective parent antibiotic. In sum, the heteromolecular hybrid dimers formed by laccase-mediated reaction had a lower, a similar or even a higher antimicrobial efficacy compared with the parent compounds (Mikolasch et al. 2006, 2007, 2008c, 2012, 2016).

Besides the in vitro antibacterial properties, the in vivo efficacy of the penicillin, cephalosporin and carbacephem hybrid dimers was tested (for comparisons please refer also to Table 1). Mice were infected with *Staphylococcus aureus* and the survival was determined after administration of the antibiotic products. Thus, for the antibiotics coupled with 2,5-dihydroxybenzoic acid derivatives (i.e. *para*-dihydroxylated), all mice survived without intoxication (Mikolasch et al. 2006, 2007). For the *β*-lactam antibiotics derivatized with *ortho*-dihydroxylated aromatic compounds 33–67% of the mice survived, whereas with the penicillin or cephalosporin used for the synthesis, all mice survived. Intoxication of mice was detected for the products (Mikolasch et al. 2008c). *Para*-hydroquinones such as methylhydroquinone or 2,3-dimethylhydroquinone used for the laccase-mediated derivatization of *β*-lactam antibiotics resulted in products which showed also in vivo activity against *S. aureus* (Mikolasch et al. 2016). Thereby the products with a penicillin part were more effective than those with a cephalosporin or carbacephem part. The penicillin hybrid dimers were also tested against multidrug-resistent *S. aureus* in this in vivo assay. The results showed a beneficial effect of the administered laccase products (Mikolasch et al. 2016).

The differences in the in vivo efficacy of the hybrid dimers in particular accompanied with cytotoxicity may be at least in part attributed to the toxicity of the respectively used laccase substrate. Thus, 2,5-dihydroxybenzoic acid and its derivatives showed a maximum of 5–14% loss of cell viability at 100 µg/ml in the cytotoxicity assay using Fl-cells (human amniotic epithelial cell line). On the contrary, *ortho*-dihydroxylated compounds such as 3-methylcatechol or alkyl-substituted *para*-hydroquinones such as 2,3-dimethyl-1,4-hydroquinone resulted in 15–25% or even 50% reduced cell viability, respectively (Mikolasch et al. 2016). Additionally, the products with 2,5-dihydroxyphenylacetic acid which is structurally similar to 2,5-dihydroxybenzoic acid showed limited in vivo efficacy. Two out of four tested hybrid dimers from a coupling with cephalosporins resulted in only 33–67% survival of mice whereas with the other two cephalosporin products all mice survived (Mikolasch et al. 2012). The dimers which resulted from a coupling of 2,5-dihydroxyphenylacetic acid to penicillins resulted also in mice survival (Mikolasch et al. 2012). Thus, further studies about the relationship between structure and efficacy are needed.

**Fig. 5:**
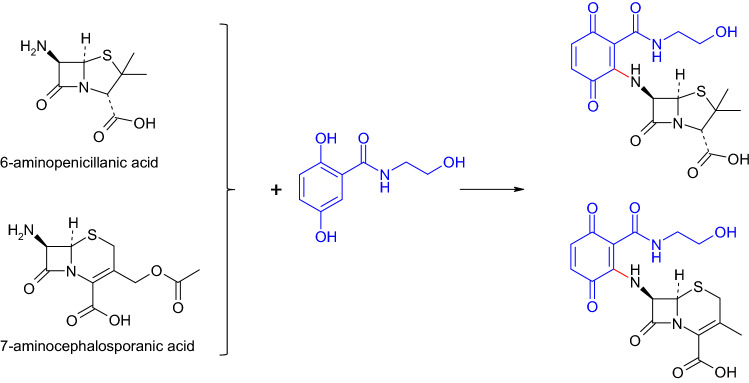
Formation of heteromolecular dimers in laccase-mediated reactions of basic structures of *β*-lactam antibiotics and 2,5-dihydroxy-*N*-(2-hydroxyethyl)-benzamide (Mikolasch et al. 2020)

To elucidate at least in part this relationship, basic structures of *β*-lactam antibiotics (6-aminopenicillanic acid, 7-aminocephalosporanic acid, 7-aminodesacetoxycephalosporanic acid) were coupled with 2,5-dihydroxybenzoic acid derivatives by laccase (Fig. 5; Mikolasch et al. 2020). The products possessed a medium antibacterial efficacy. In contrast, to the initially used penicillanic and cephalosporanic acids which were inactive against the tested microorganisms. However, the efficacy was mostly lower than described for the derivatives of amoxicillin or cefadroxil formed by laccase. This may be attributed to the initially antibacterial efficacy of the parent antibiotics amoxicillin and cephalosporin. Furthermore, Mikolasch et al. (2020) assumed that the quinonoid ring of the oxidized laccase substrate (instead of the phenolic ring in amoxicillin and cefadroxil, please compare Table 2 and Fig. 5) on the *β*-lactam ring of the C–N dimer may result in a reduced protection of this ring.

Thus, degrading enzymes such as *β*-lactamases could cleave the *β*-lactam ring more easily and the antibiotic may no longer inhibit bacterial peptidoglycan synthesis (Mikolasch et al. 2020). However, *β*-lactamase stability may be a favourable property of novel antibiotics. Thus, the synthesized penicillin, cephalosporin and carbacephem hybrid dimers revealed that it is possible to produce compounds by means of laccase that possess a higher stability against *β*-lactamase (Mikolasch et al. 2006, 2016). Additionally, the log D value of all products with penicillins and 2,5-dihydroxybenzoic acid derivatives was also higher compared with the parent penicillins ampicillin and amoxicillin (Mikolasch et al. 2006). The products possessed log D values between 1.2 and 2.9 whereas that the parent antibiotics showed negative log D values. This results in a potentially good absorption of the products by the gastrointestinal tract and thereby allows peroral administration. The individual test results demonstrate that laccase-mediated derivatization of antibiotics can be used to produce new substances that may have a similar or even better effect to the parent compounds.

### Sulfonamides

**Fig. 6:**
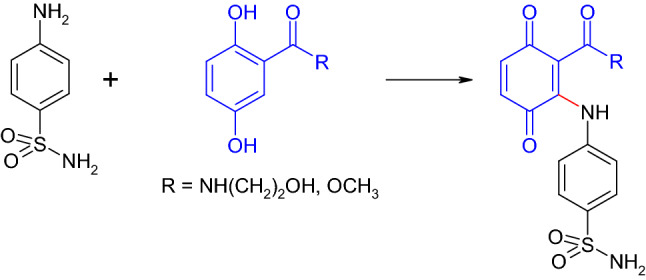
Formation of heteromolecular dimers in the laccase-mediated reaction of sulfanilamide and 2,5-dihydroxybenzoic acid derivatives (Mikolasch and Hahn 2021)

The promising antimicrobial effect in vivo for the *β*-lactam antibiotics derivatized with *para*-dihydroxylated aromatic compounds led to further research efforts. Thus, antibiotics with sulfonamide or sulfone structures were coupled with 2,5-dihydroxybenzene derivatives by laccase of *Trametes* spec. (Mikolasch and Hahn 2021). The reactions resulted in the formation of different hydroquinonoid and quinonoid dimers and trimers. The two tested sulfanilamide derivatives (Fig. 6) showed low to medium antibacterial efficacy. 4′-sulfonamide-2,4-diaminoazobenzene (trade name: Prontosil) was the first sulfonamide antibiotic (Domagk 1935). The antibacterial effect of sulfonamides and sulfones relies on the inhibition of folic acid synthesis which is essential for the formation of nucleic acid (Sköld 2000; Zhu and Stiller 2001; Wainwright and Kristiansen 2011).

**Fig. 7:**
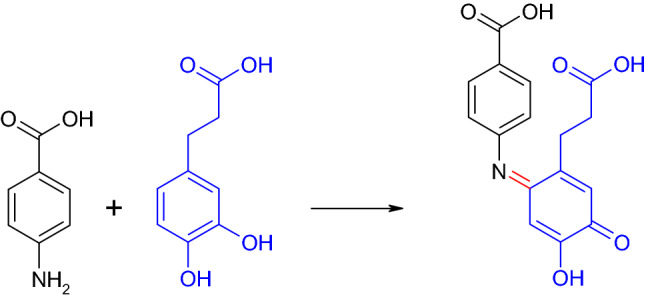
Formation of a heteromolecular dimer in the laccase-mediated reaction of 4-aminobenzoic acid and 3-(3,4-dihydroxyphenyl)-propionic acid (Mikolasch et al. 2002; Niedermeyer et al. 2005)

4-Aminobenzoic acid is a structural part of Prontosil as well as candicidin D. The latter is a heptaene macrolide with antifungal properties (Lechevalier et al. 1953; Hamilton-Miller 1973). The 4-aminobenzoic acid itself possesses an antibacterial activity, for example, against *Escherichia coli* that was attributed at least in part to a destruction of the outer cell membrane (Eagon and McManus 1990; Richards and Xing 1992a, b; Richards et al. 1995). Furthermore, 4-aminosalicylic acid (4-amino-2-hydroxybenzoic acid) is used as antimycobacterial drug (Zheng et al. 2013). The laccase-mediated derivatization of 4-aminobenzoic acid with *para*-dihydroxylated aromatic compounds was described repeatedly (Manda et al. 2005; Niedermeyer et al. 2005; Niedermeyer and Lalk 2007; Mikolasch et al. 2008b). These reactions resulted in C–N dimers and trimers. In contrast, quinone-imine formation was described for the reaction of the *ortho*-dihydroxylated substance 3-(3,4-dihydroxyphenyl)-propionic acid with 4-aminobenzoic acid (Fig. 7; Mikolasch et al. 2002; Niedermeyer et al. 2005).

### Oligosaccharides

**Fig. 8:**
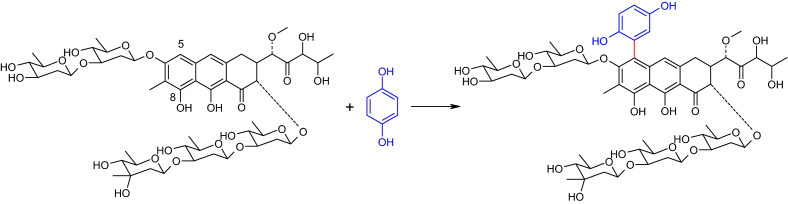
Formation of a heteromolecular dimer in the laccase-mediated reaction of mithramycin and hydroquinone (Anyanwutaku et al. 1994)

Anyanwutaku et al. (1994) described the laccase-catalyzed reaction of mithramycin (also known as plicamycin), an antitumor antibiotic and hydroquinone (Fig. 8). The reaction was mediated by the laccase of *Polyporus anceps* and resulted in the formation of a heteromolecular dimer. The product was also formed in the non-enzymatic catalyzed reaction with benzoquinone. Structural analyses of the products revealed C–C bond formation between the hydroquinone and the aglycone of mithramycin. The authors assumed that not only the hydroquinone but also the mithramycin, i.e. both compounds were oxidized by the laccase. The resulting phenoxy radical in position C-8 of the mithramycin isomerized to a C-5 carbon radical and then coupled with the semiquinone radical formed from hydroquinone. The resulting heteromolecular dimer showed no effect against a leukemia cell line. It was suggested that due to the blocking of the C-5 position, the formation of reactive radicals or binding to DNA was prevented.

It has to be mentioned, that hydroquinone and benzoquinone itself have antibacterial activity (Tran et al. 2004). Furthermore, compounds with hydroquinonoid and benzoquinonoid structures have been shown previously to possess antimicrobial efficacy. Thus, ganomycins are antibacterial farnesyl hydroquinones isolated from fungi (Mothana et al. 2000). The aminoquinones mitomycin C (Takada et al. 1977; Bradner 2001) and streptonigrin (Bolzán and Bianchi 2001; Bringmann et al. 2008) combine antibiotic and antitumour activities. The synthesis of mitomycin analogs has been described for a two-enzyme system consisting of laccase and lipase (Zhang et al. 2020).

### Aminoglycosides

**Fig. 9:**
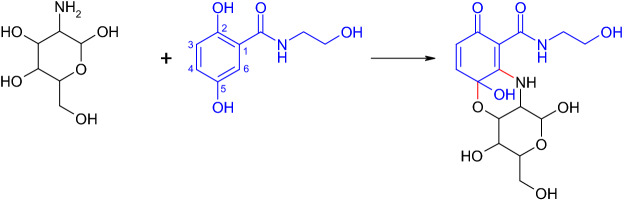
Formation of a heteromolecular dimer in the laccase-mediated reaction of glucosamine and 2,5-dihydroxy-*N*-(2-hydroxyethyl)-benzamide (Mikolasch et al. 2022)

Aminoglycosides bind on the 30S subunit of ribosomes and thereby impaire the formation of proteins (Mingeot-Leclercq et al. 1999). Aminoglycoside antibiotics and glucosamine were derivatized with 2,5-dihydroxy-*N*-(2-hydroxyethyl)-benzamide (Mikolasch et al. 2022). The products resulted from a laccase-mediated reaction of the amino group with the 2,5-dihydroxy-*N*-(2-hydroxyethyl)-benzamide and with the carbonyl group at C-5 position (Fig. 9). The glucosamine derivative showed in accordance with the reactants no effect against the tested bacteria whereas the products with aminoglycoside antibiotics (kanamycin, gentamycin, tobramycin) possessed a medium to high antibacterial efficacy. The product with gentamicin inactivated the multidrug-resistant *S. epidermidis* whereas gentamicin was inactive against this bacterium (Mikolasch et al. 2022). Gentamicin as well as kanamycin were mixtures of different structures due to various substituents resulting in product mixtures with 2,5-dihydroxy-*N*-(2-hydroxyethyl)-benzamide. The in vivo assays in mice resulted in a similar efficacy of the aminoglycoside derivatives than the antibiotic used for the synthesis. In addition, no toxicity was determined (Mikolasch et al. 2022).

### Corollosporines

**Fig. 10:**
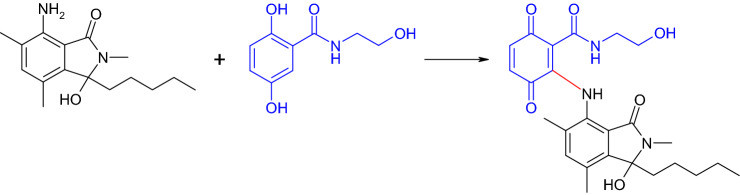
Formation of a heteromolecular dimer in the laccase-mediated reaction of a *N*-analogous corollosporine and 2,5-dihydroxy-*N*-(2-hydroxyethyl)-benzamide (Mikolasch et al. 2008a)

Corollosporines possesses a phthalide structure and antimicrobial properties, e.g. against *Staphylococcus aureus* (Liberra et al. 1998). *N*-analogous corollosporine derivatives were subjected to laccase-catalyzed reactions with *para*- and *ortho*-dihydroxylated aromatic compounds, whereby only products with *para*-dihydroxylated aromatics were isolated (Fig. 10; Mikolasch et al. 2008a; Mikolasch 2019). All parent substances had no or only low antibacterial activity, whereas the newly synthesized products showed an inhibiting efficacy in particular against various gram-positive bacteria including multidrug-resistant *Staphylococcus* strains (Mikolasch et al. 2008a).

### Azoles and morpholines

The azoles and morpholines used in medicine or agriculture act on the sterol biosynthesis of fungi (Vanden Bossche et al. 1984; Hitchcock et al. 1989; Mercer 1991; Ziogas et al. 1991), although different targets have already been described between these two groups. Azoles inhibit mainly cytochrome P450-dependent 14α-demethylase (Vanden Bossche et al. 1984; Hitchcock et al. 1989; Francois et al. 2005), whereas morpholines act on sterol Δ^8^ → Δ^7^-isomerase and Δ^14^-reductase (Baloch et al. 1984; Baloch and Mercer 1987; Mercer 1991; Polak 1992). In both cases, the formation of ergosterol (a major component of the fungal cell membrane) from lanosterol is prevented, leading to disruption of membrane structure and to cell death (Steel et al. 1989; Ziogas et al. 1991).

**Fig. 11:**
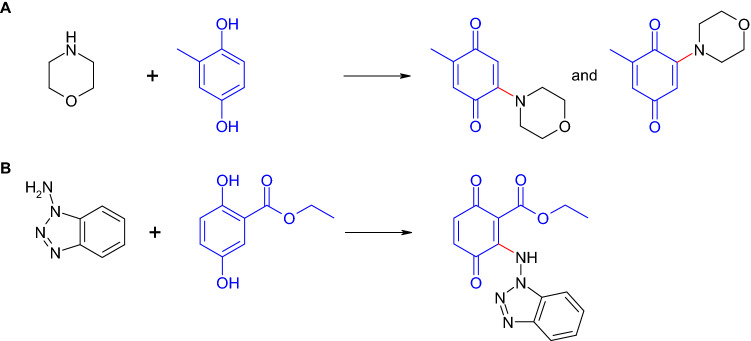
Formation of heteromolecular dimers in the laccase-mediated reactions of **A** morpholine with methylhydroquinone (Hahn et al. 2009) and **B** 1-aminobenzotriazole with 2,5-dihydroxybenzoic acid ethyl ester (Hahn et al. 2010b)

The laccase-mediated reaction of antifungal substances or its basic structures such as azoles and morpholines with *para*-dihydroxylated aromatic compounds resulted in quinonoid hybrid dimers and trimers (Hahn et al. 2009, 2010b, 2014). Additionally, the reaction of morpholine with 2,5-dihydroxy-*N*-(2-hydroxyethyl)-benzamide resulted in a hydroxylated dimer (Hahn et al. 2009). The azole as well as the morpholine hybrid dimers (Fig. 11) showed a low to medium growth inhibition of bacteria and the yeast *Candida maltosa* (Hahn et al. 2009, 2010b). In contrast, the hydroxylated morpholine dimer and the morpholine trimers possessed no antimicrobial activity (Hahn et al. 2009). The efficacy of the dimers against bacteria and fungi suggested a broad-spectrum application but may also pointed to a general cytotoxicity. This assumption is not entirely conclusive (as discussed also previously for the *β*-lactams) because the trimers showed cytotoxicity against a cancer cell line (HTB-9) but no antimicrobial activity (Hahn et al. 2009). Thus, the cytotoxicity may play an important role for a product synthesized by laccase but it is not the only factor that defines antimicrobial properties. The studies showed that products formed by laccase-mediated reactions may possess a lower, a similar or a higher antimicrobial efficacy.

Different azoles were coupled with hydroquinone (Bhalerao et al. 1994) or 2,5-dihydroxybenzoic acid and its derivatives (Hahn et al. 2010a). The cyclization reactions included C–S, C–N, C=N, and C=O bond formations and resulted in thiadiazines (Bhalerao et al. 1994), cycloheptenes, cyclooctenes, diazaspiro cyclohexenes and phenazines (Hahn et al. 2010a). The thiadiazines and cycloheptenes were produced with yields of 83–95% and 11–71%, respectively. Especially for the cycloheptenes a pharmaceutical application may be conceivable. Thus, cycloheptenes are structurally related to diazepines which have anticonvulsant, antianxiety (Childress and Gluckman 1964; Goodkin and Kapur 2009), antitumor (Andreyanova et al. 1998), antimicrobial, anthelmintic (Kumar and Joshi 2009) and anti-HIV (Görlitzer et al. 1995) activities.

**Fig. 12:**
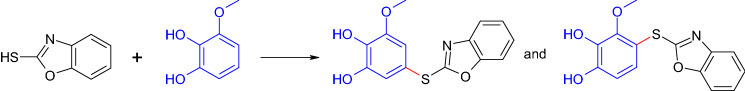
Formation of heteromolecular dimers in the laccase-mediated reaction of 2-mercaptobenzothiazole and 3-methoxycatechol (Abdel-Mohsen et al. 2014)

The group of Beifuss et al. studied the derivatization of azoles as well as the synthesis of structures with azole part by laccase-mediated reaction. Thus, Abdel-Mohsen et al. (2014) described the laccase-mediated formation of thioethers from benzoxazoles and catechols (Fig. 12). Such compounds were previously synthesized by electrooxidative Michael reaction and tested for the antimicrobial and antioxidative properties (Adibi et al. 2011). One of these products (Fig. 12—product left: 2-mercaptobenzothiazole in *meta*-position to the methoxy group of the catechol) was also part of the laccase study and showed activity against gram-positive and gram-negative bacteria as well as *Candida albicans* (mostly clinical isolates). Additionally, an antioxidative property of this product was determined (Adibi et al. 2011). Previous studies comprised laccase-catalyzed domino/cascade reactions and resulted in the formation of benzimidazoles (Leutbecher et al. 2011) and pyrimidobenzothiazoles with yields of 15–99% (Abdel-Mohsen et al. 2013, 2017).

### Phenoxazinones, phenoxazines, phenazines, phenothiazinones, phenothiazines

**Fig. 13:**
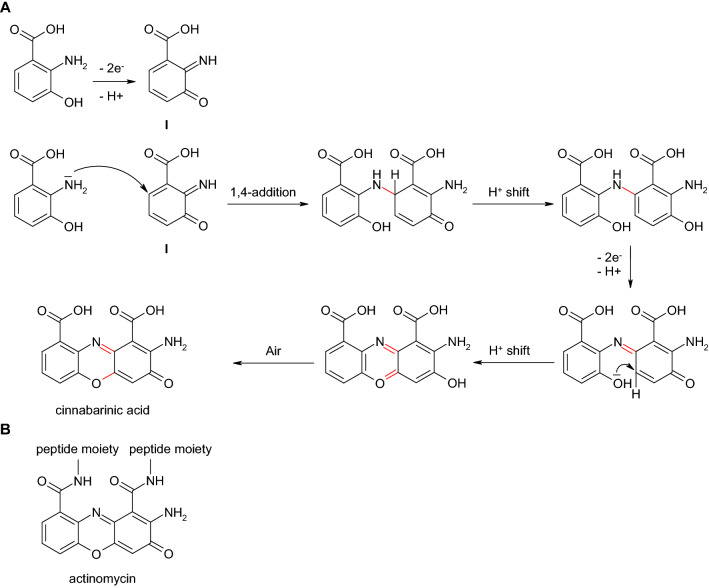
**A** Formation of cinnabarinic acid in the laccase-mediated reaction of 3-hydroxyanthranilic acid (2-amino-3-hydroxybenzoic acid; Eggert et al. 1995); **B** chemical structure of actinomycin (Freeman et al. 1993)

Phenoxazinone synthesis was described for members of the genus *Pycnoporus* which produce orange-red pigments that are responsible for the characteristic color of the fruiting bodies. The pigments of the phenoxazinone type include cinnabarin, tramesanguin, and cinnabarinic acid (Gripenberg 1951, Sullivan and Henry 1971). Because of this, *Pycnoporus cinnabarinus* is also called "cinnabar-red or vermilion polypore". This naturally occuring laccase-mediated synthesis of phenoxazinones was employed for the in vitro production. Eggert et al. (1995) used the laccase of *Pycnoporus cinnabarinus* and 3-hydroxyanthranilic acid (2-amino-3-hydroxybenzoic acid), which is formed in nature by the fungus itself, for the synthesis of cinnabarinic acid (2-amino-3-oxo-3*H*-phenoxazine-1,9-dicarboxylic acid; Fig. 13A). Thereby, an *ortho*-quinone imine (**I**) was formed by two steps of one-electron oxidations which undergo Michael addition through the nucleophilic amino group of the second aminophenol (in this case 3-hydroxyanthranilic acid) molecule. After additional laccase-catalyzed two electron oxidations an intramolecular Michael addition resulted in the phenoxazine (Eggert et al. 1995; Bruyneel et al. 2012; Sousa et al. 2014).

Cinnabarinic acid is probably formed as a product of antioxidative reactions in the course of the radical scavenging property of 3-hydroxyanthranilic acid. Eggert (1997) tested the antibacterial activity of cinnabarinic acid due to the structural similarity to actinomycin (Fig. 13B), a peptide antibiotic (Waksman et al. 1946). Compared to 3-hydroxyanthranilic acid, the cinnabarinic acid showed a different, i.e. an antibacterial efficacy. This example proves that by means of the laccase-catalyzed reaction, products can be formed which have different effects than the parent substances used for the synthesis. The culture supernatant separated and concentrated from the mycelium of the fungus contained large amounts of cinnabarinic acid and showed similar antibacterial activity compared to the cinnabarinic acid produced in vitro by laccase. In general, the inhibitory effect against gram-positive bacteria especially *Streptococcus* species was greater than for gram-negative bacteria. On the contrary, no cinnabarinic acid and no antibacterial activity was determined in a laccase deletion mutant of *Pycnoporus cinnabarinus* although 3-hydroxyanthranilic acid was formed (Eggert 1997). Interestingly, similar enzymes mediate the synthesis of cinnabarinic acid and actinomycin. Thus, the phenoxazinone synthase of *Actinomyces* (*Streptomyces) antibioticus*, responsible for actinomycin D synthesis, belongs as the fungal laccase to the blue copper oxidases (Barry et al. 1989; Freeman et al. 1993; Eggert et al. 1996). Actinomycin, in addition to an antibacterial effect, also has a cytotoxic effect, which result in a classification as antitumor antibiotic. The antitumor effect of actinomycin is attributed to the inhibition of DNA transcription. Actinomycin binds to DNA and thus prevents RNA synthesis (Goldberg et al. 1962; Reich et al. 1962; Morioka et al. 1985). For cinnabarinic acid also a cytotoxic activity has been described. Cinnabarinic acid showed a tenfold higher inducing activity of apoptosis towards thymocytes than 3-hydroxyanthranilic acid (Hiramatsu et al. 2008).

**Fig. 14:**
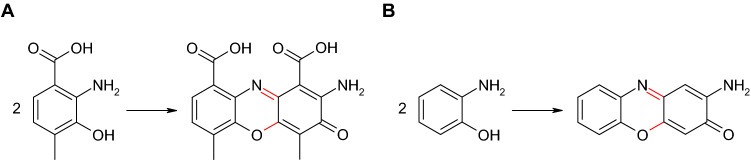
**A** Formation of actinocin in the laccase-mediated reaction of 4-methyl-3-hydroxyanthranilic acid (2-amino-3-hydroxy-4-methylbenzoic acid; Osiadacz et al. 1999); **B** formation of questiomycin in the laccase-mediated reaction of 2-aminophenol (Giurg et al. 2007)

In addition to the synthesis of cinnabarinic acid from 3-hydroxyanthranilic acid, further phenoxazinone derivatives with potentially advantageous properties for pharmaceuticals were formed by laccase-mediated reactions (Osiadacz et al. 1999; Bruyneel et al. 2008, 2009, 2010; Forte et al. 2010; Sousa et al. 2014, 2018). For example, Osiadacz et al. (1999) described laccase-catalyzed formation of actinocin (2-amino-4,6-dimethyl-3-oxophenoxazine-1,9-dicarboxylic acid) from 4-methyl-3-hydroxyanthranilic acid (2-amino-3-hydroxy-4-methylbenzoic acid; Fig. 14A) similar to actinomycin (Barry et al. 1989). The synthesis of questiomycin as well as cinnabarinic acid and actinocin mediated by laccase or peroxidase or chemical substances has also been shown (Fig. 14B; Giurg et al. 2007). Bruyneel et al. (2008, 2009, 2010) produced phenoxazinone derivatives that were characterized by higher polarity and consequently would be more suitable for biological applications. Therefore, chemical sulfonation of 2-aminophenol was followed by the formation of a phenoxazinone with the laccase of *Trametes versicolor*.

The antimicrobial efficacy of the phenoxazinones synthesized by laccase may be dependent on the substituents. Thus, no antibacterial activity was determined (under the chosen conditions) for the phenoxazinone synthesized from 3-amino-4-hydroxybenzenesulfonic acid (Polak et al. 2016). The authors proposed that the lack of substituents such as carboxy, methoxy or cyclic pentapeptides could be responsible for this effect.

In contrast to the *ortho*-substituted compounds, Shaw and Freeman detected phenazines (Shaw and Freeman 2004) during laccase-mediated reactions of the *para*-phenylenediamine 2,5-diaminobenzenesulfonic acid. Additionally, Sousa et al. (2014) described also the synthesis of phenazines as well as phenoxazinones and phenoxazines using different substituted *ortho*-aminophenols as well as *ortho*- and *para*-phenylenediamines. In further experiments, the spectrum of phenoxazinones and phenazines was extended (Sousa et al. 2018). For both studies, the bacterial CotA-laccase was used.

**Fig. 15:**
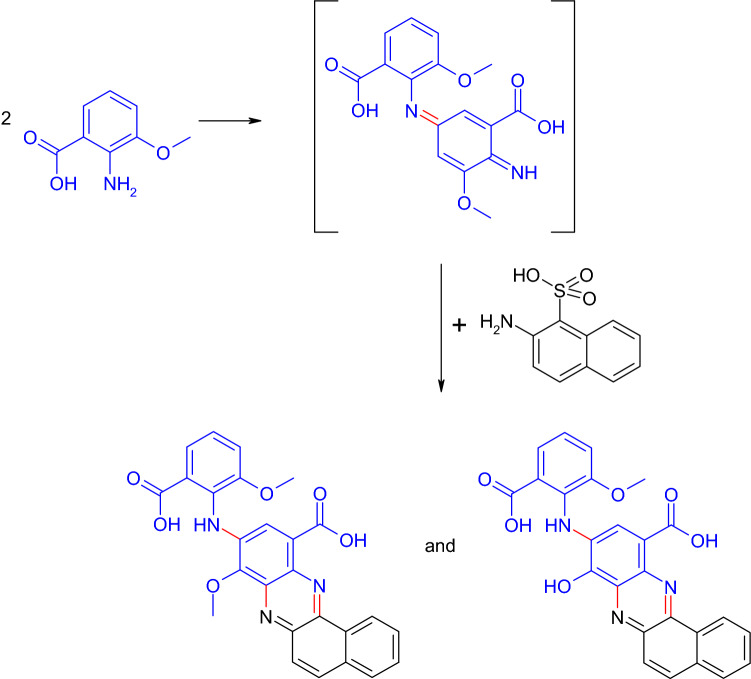
Formation of phenazines in the laccase-mediated reaction of 2-aminomethoxybenzoic acid with 2-aminonaphthalene-1-sulfonic acid (Polak et al. 2020)

Bruyneel et al. (2012) expanded the concept of phenoxazinone formation by a coupling of two different *ortho*-aminophenols resulting in non-symmetrical phenoxazinones. Products that are more complex were synthesized. Thus, phenazines were formed by a homomolecular coupling product that reacted with another partner (Fig. 15; Polak et al. 2020). The compounds possessed antimicrobial and antioxidative properties (Polak et al. 2020). These examples confirmed that the synthesis of cyclization products is not only possible with one reactant in the laccase assay.

**Fig. 16:**
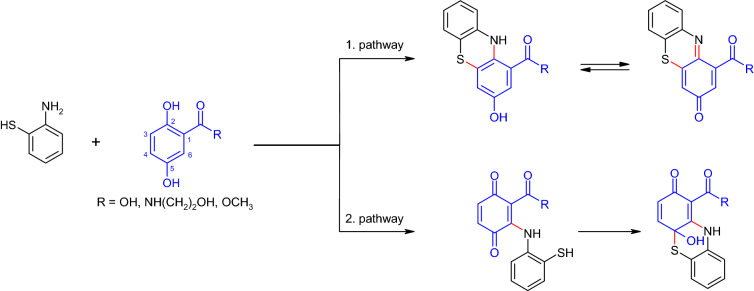
Formation of heteromolecular products by two pathways in the laccase-mediated reaction of 2-aminothiophenol and 2,5-dihydroxy-*N*-(2-hydroxyethyl)-benzamide (Hahn et al. 2020)

Indeed, the formation of hybrid dimers by cyclization reactions between dihydroxylated aromatic compounds and a partner with two nucleophilic substituents has been described. Again 2,5-dihydroxybenzoic acid and its derivatives were coupled with *ortho*-substituted arylamines or arylthiols (Hahn et al. 2020). The formed phenoxazines, phenazines and phenothiazines may have different efficacies. Thus, various properties have been described for these substance groups, e.g. antimicrobial (Waksman et al. 1946; Geiger et al. 1988; Bansode et al. 2009) and antitumor (Takahashi et al. 1986; Zhao et al. 2016; Zhang et al. 2017) activity. Remarkably, for the phenothiazines multidrug resistance reverting activity was determined (Kolaczkowski et al. 2003; Bisi et al. 2008). The formation of phenothiazinones, the oxidation products of phenothiazines, has been described for the laccase-mediated reaction of 2-aminothiophenol with *para*-hydroquinones, *para*-quinones (Marcinkeviciene et al. 2013; Cannatelli and Ragauskas 2016) or 2,5-dihydroxybenzoic acid derivatives (Hahn et al. 2020). Cannatelli and Ragauskas (2016) described the amination at the carbonyl group of the quinone with cyclization via the thiol group. Hahn et al. (2020) showed two pathways for phenothiazine/phenothiazinone synthesis. The first one was proposed as described by Cannatelli and Ragauskas (2016) with a reaction of the amino group on the C-2 of the oxidized 2,5-dihydroxybenzoic acid derivative followed by a ring closure on C-3. The second pathway comprised a intermolecular Michael addition of the amino group with the C-6 of the oxidized 2,5-dihydroxybenzoic acid derivative and subsequent intramolecular 1,2-addition. Limited experimental data showed an antimicrobial efficacy of the end product depicted in Fig. 16 (R = NH(CH_2_)_2_OH, second pathway; data not shown).

The phenoxazine formation instead of expected phenoxazinones (Bruyneel et al. 2009; Sousa et al. 2014) as well as the detection of phenothiazines and phenothiazinones in one reaction (Hahn et al. 2020) could be attributed to a hydrolysis of the imine function possibly during product purification or different mechanisms during the synthesis pathway (Bruyneel et al. 2009).

## Further compounds

The laccase-mediated reactions can result in products, which possess antioxidative as well as additional properties. Thus, the reactions of luteolin or isorhamnetin (flavons of plants) resulted in the formation of dimers that showed a higher antioxidative property compared to the respective reactant (Aruwa et al. 2021, 2022). The antibacterial efficacy was also higher and included inactivation of methicillin-resistant *Staphylococcus aureus* (Aruwa et al. 2021, 2022). Furthermore, the synthesis of homo- and hetermolecular products with anilines resulted in compounds effective against the phytopathogenic fungus *Botrytis cinerea* (Campos et al. 2016; Mendoza et al. 2016; Castro et al. 2019). Thus, the laccase-mediated reaction of *p*-chloroaniline led to the formation of 4,4’-biphenyldiamine which showed antioxidative as well as antifungal activities (Campos et al. 2016). The coupling of anilines with syringic acid (4-hydroxy-3,5-dimethoxybenzoic acid) resulted in hybrid dimers with quinone-imine structures. The products possessed a higher efficacy against *Botrytis cinerea* than the parent substances and may be used as pesticides in agriculture. The antifungal activity of the dimers is probably based on a destructive effect on the fungal cell wall possibly caused by the interaction with enzymes involved in chitin and glucan synthesis (Mendoza et al. 2016; Castro et al. 2019).

### Influence of reaction conditions

In general, multiple factors should be taken into account for the design of laccase-mediated reactions. This includes e.g. pH-value of the reaction medium, redox potential of the laccase and the substrate as well as accessibility of the substrate towards the catalytic centre. Additionally, the reaction temperature can be varied according to the stability of the enzyme, whereby room temperature is more favoured with regard to the principles of green chemistry (Anastas and Warner 1998).

The pH-value of the buffer solution should comply with the pH-optimum of the enzyme, whereby the kind of substrate influences the determination of the pH-optimum and may result in differences. Nevertheless, the reaction assays with laccase of *Pycnoporus cinnabarinus* (*Pc*l) or *Trametes* spec. (*Ts*l) were performed with a buffer pH of 5 (Mikolasch et al. 2006, 2007; Castro et al. 2019) whereas for *Myceliophthora thermophila* laccase (*Mt*l) the buffer pH was 7 (Mikolasch et al. 2007). As mentioned above, laccases differ in the redox potential. Thus, *Ts*l and *Pc*l possess a high potential, while *Mt*l has a low redox potential. Experiences with these laccases resulted in the conclusion, that the synthesis of antimicrobial compounds from aminated substances and simple *para*-hydroquinones such as hydroquinone itself or methylhydroquinone proceed better (in regard of reaction rate and stable product formation) with *Mt*l at pH 7 than with *Ts*l or *Pc*l at pH 5. In opposite, for reactions with 2,5-dihydroxybenzoic acid and its derivatives *Ts*l or *Pc*l is a good choice (Mikolasch et al. 2007; Hahn et al. 2014). Nevertheless, the oxidation of compounds is also dependent on the kind and amount of substituents on the aromatic ring.

In most cases, the reaction medium, for the synthesis of antimicrobial compounds, was only buffer (Mikolasch et al. 2008c; Mikolasch and Hahn 2021) but also solvents are conceivable, e.g. in order to solve a hydrophobic reactant. Different amounts of water-miscible organic solvents in buffer -max. 10%- (Abdel-Mohsen et al. 2014; Hahn et al. 2020) or even 50% (Castro et al. 2019) were described. The synthesis of the phenoxazinone actinocin yielded 72% in an assay containing 60% acetonitrile in buffer (Osiadacz et al. 1999) whereas the dimer formation of 2,5-dihydroxybenzoic acid derivative and an azole was diminished with methanol concentrations higher than 25% (Hahn et al. 2010b). Additionally, also biphasic media has been described for the synthesis of biologically active substances (Campos et al. 2016). Regarding the selection of „green “ solvents the reader is referred to (Prat et al. 2016). The adaption of the reaction environment is defined by the term “medium-engineering”.

Furthermore, a variation of the reactant concentration is also possible and may enhance the formation of a particular product. An increase of the catechol concentration from 1 to 1.25 mmol compared to the reaction partner 2-mercaptobenzothiazole resulted in a 16% higher yield of the hybrid dimer (Abdel-Mohsen et al. 2014). The laccase-mediated synthesis of dimers consisting of syringic acid and different anilines was most effective for an equal amount of the reactants (0.1 mmol) than with an excess of one of the two partners (Castro et al. 2019). But, in case of a targeted synthesis of trimers consisting e.g. of one molecule hydroquinone and two molecules morpholine an excess of morpholine should be considered (Hahn et al. 2009). Nevertheless, a higher amount of one partner bears the risk for residual amounts of non-transformed compounds or impurities due to homomolecular products which may be problematic for product purification. In terms of product isolation also an increase of both reaction partners is applicable (Mikolasch et al. 2008a; Mikolasch and Hahn 2021).

Thus, the enzyme selectivity, the transformation rates, and the resulting product pattern can be adapted and optimized to the targeted synthesis using different reaction conditions.

## Concluding remarks

The shown examples confirm the suitability of the enzyme laccase for the synthesis and derivatization of antimicrobial compounds. In particular, heteromolecular reactions open up the possibility to couple different substances, which may result in an increased biological efficacy or the development of a new property. The laccase-mediated formation of compounds with antimicrobial efficacies at least similar or even higher compared to the parent substances (Table 1; Mikolasch et al. 2006, 2007, 2008a, c, 2012, 2016, 2020, 2022; Hahn et al. 2009, 2010b; Mikolasch and Hahn 2021), in particular compounds effective against multidrug-resistant microorganisms should be part of ongoing research to overcome the dissemination and increasing amount of multidrug-resistant microorganisms. In addition, the synthesis of antimicrobial active products from *N*-analogous corollosporine derivatives, sulfanilamide, 1-aminobenzotriazole as well as penicillanic or cephalosporanic acids with 2,5-dihydroxybenzoic acid derivatives—which were inactive (at the tested concentration) against almost all tested microorganisms—support the utilization of laccase for the production of novel antimicrobial substances (Mikolasch et al. 2008a, 2020; Hahn et al. 2010b; Mikolasch and Hahn 2021).

Despite limited data on susceptibility tests against bacteria and fungi, Mikolasch et al. provided not only in vitro but also in vivo analyses for the products formed by laccase-mediated reactions (Mikolasch et al. 2006, 2007, 2008c, 2012, 2016, 2022). The experiments showed promising results for hybrid dimers (C–N or C–N C–O dimers) of penicillins or glucosamine coupled with *para*-dihydroxylated aromatic compounds. Nevertheless, efforts are required for the characterization of products, formed by laccase-mediated reactions, to determine the respective biological efficacy. This will allow an analysis of the relationship between molecular structure and efficacy leading to a more targeted reaction design with development of valuable compounds for pharmaceutical applications. Thereby, not only the selection of reactants is of importance but also the influence of reaction parameters such as pH-value or reactant concentration should be analyzed. The suitability of laccases with improved properties (e.g. increased activity) generated by protein-engeneering should also be examined to increase the product yield and facilitate a scale up which is indispensable for the industrial production of pharmaceuticals or pesticides. In addition, the described laccase-catalyzed processes may also be useful for the antimicrobial functionalization of surfaces (grafting) such as wood (Kudanga et al. 2008) or textiles (Schroeder et al. 2007; please refer also to reviews Nyanhongo et al. 2010 and Slagman et al. 2018). Thus, for the phenoxazinones and other products formed by laccase-mediated syntheses an application in dying industry was suggested and in part already patented (Polak and Jarosz-Wilkolazka 2012). Coated or dyed textile fibers may even possess antimicrobial efficacy (Schroeder et al. 2007; Polak et al. 2020). The production of fibres with such additional properties is a further research and application possibility for the enzyme laccase. The environmentally friendly laccase reactions are valuable in green chemistry for the synthesis and derivatization of antimicrobial compounds. More efforts are needed to motivate stakeholders in particular in medicine, chemical and pharmaceutical companies to see the great potential of products that were synthesized by laccase.
